# The complete mitochondrial genome of *Thalamita sima* (Decapoda: Portunidae)

**DOI:** 10.1080/23802359.2018.1483777

**Published:** 2018-06-26

**Authors:** Shengping Zhong, Yanfei Zhao, Qin Zhang

**Affiliations:** Key Laboratory of Marine Biotechnology, Guangxi Institute of Oceanology, Beihai, China

**Keywords:** Mitochondrial genome, *Thalamita sima*, Decapoda

## Abstract

*Thalamita* is one of most important genera of Portunidae. However, the systemically classification and taxonomic studies have so far been limited. In this study, we report the complete mitochondrial genome sequence of *T. sima*. The mitogenome has 15,831 base pairs (71.7% A + T content) and made up of total of 37 genes (13 protein-coding, 22 transfer RNAs and 2 ribosomal RNAs), and a putative control region. This study was the second available complete mitogenomes of *Thalamita* and will provide useful genetic information for future phylogenetic and taxonomic classification of *Thalamita*.

*Thalamita* is one of the most important genera of Portunidae, which contains the most diverse species after *Portunus* among the swimming crabs (Portunidae) (Tan et al. 2016). There are about 90 species belonging to *Thalamita*, however, systemically classifications on them have not been done and misidentifications have been occasionally reported (Spiridonov 2017). In spite of its economic and ecological significance, adequate genetic information about the genus is still missing (Tan et al. 2016; Saher et al. 2018). Here, we report the second complete mitochondrial genome sequence of genus *Thalamita*, which will provide a better insight into phylogenetic assessment and taxonomic classification.

A tissue sample of *T. sima* was collected from GuangXi province, China (Beihai, 21.445126N, 109.250713 E), and the whole body specimen (#GQ0264) were deposited at Marine biological Herbarium, Guangxi Institute of Oceanology, Beihai, China. The total genomic DNA was extracted from the muscle of the specimens using an SQ Tissue DNA Kit (OMEGA, Guangzhou, China) following the manufacturer’s protocol. DNA libraries (350 bp insert) were constructed with the TruSeq NanoTM kit (Illumina, San Diego, CA, USA) and were sequenced (2 × 150 bp paired-end) using HiSeq platform at Novogene Company, China. Mitogenome assembly was performed by MITObim (Hahn et al. 2013). The complete mitogenome of *T. crenata* (GenBank accession number: NC_024438) was chosen as the initial reference sequence for MITObim assembly. Gene annotation was performed by MITOS (Bernt et al. 2013).

The complete mitogenome of *T. sima* was 15,831 bp in length (GenBank accession number: MG840650), and containing the typical set of 13 protein-coding, 22 tRNA and 2 rRNA genes, and a putative control region. The overall base composition of the mitogenome was estimated to be A 35.3%, T 36.4%, C 17.6% and G 10.7%, with a high A + T content of 71.7%, which is similar, but slightly higher than *Orithyia sinica* (69.5%) (Zhong et al. 2018). The gene order in *T. sima* is highly similar to that found in *T. crenata*, which indicated close relationship between *T. sima* and *T. crenata*. The result of phylogenetic tree of 11 species (including other 10 species from family Portunidae in NCBI) also supported the close relationship between *T. sima* and *T. crenata* (Figure 1), as they shared the same clade with the highest bootstrap value. The complete mitochondrial genome sequence of *T. sima* was the second sequenced mitogenomes within the genus *Thalamita*, which will contribute to further comparative and evolutionary mitogenome studies of the genus *Thalamita*, and related genera.

**Figure 1.**
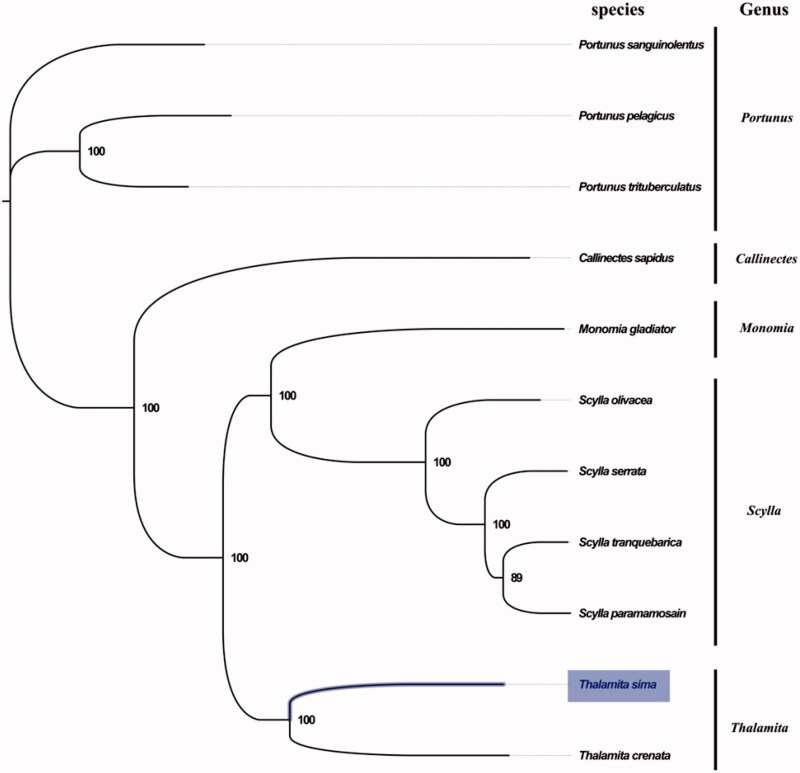
Phylogenetic tree of 11 species in family Portunidae. The complete mitogenomes are downloaded from GenBank and the phylogenic tree is constructed by maximum-likelihood method with 100 bootstrap replicates. The bootstrap values were labelled at each branch nodes. The gene's accession number for tree construction is listed as follows: *Portunus sanguinolentus* (NC_028225), *Portunus pelagicus* (NC_026209), *Portunus trituberculatus* (NC_005037), *Callinectes sapidus* (NC_006281), *Monomia gladiator* (NC_037173), *Scylla olivacea* (NC_012569), *Scylla serrata* (NC_012565), *Scylla tranquebarica* (NC_012567), *Scylla paramamosain* (NC_012572) and *Thalamita crenata* (NC_024438).

## References

[CIT0001] Bernt M, Donath A, Jühling F, Externbrink F, Florentz C, Fritzsch G, Pütz J, Middendorf M, Stadler PF 2013 MITOS: improved de novo metazoan mitochondrial genome annotation. Mol Phylogene Evol. 69:313–319. 10.1016/j.ympev.2012.08.023 22982435

[CIT0002] Hahn C, Bachmann L, Chevreux B 2013 Reconstructing mitochondrial genomes directly from genomic next-generation sequencing reads—a baiting and iterative mapping approach. Nucl Acids Res. 41:e129. 23661685 10.1093/nar/gkt371 PMC3711436

[CIT0003] Saher NU, Naz F, Siddiqa SM, Khan MM, Kamal M 2018 Re description and molecular identification of Thalamita danae (Stimpson 1858) (Decapoda: Brachyura: Portunidae) based on fresh material from the coastal waters, Pakistan. Mitochondrial DNA Part B. 3:332–335. 10.1080/23802359.2018.1438855 PMC7800256 33490505

[CIT0004] Spiridonov VA 2017 Two new species of Latreille, 1829 (Decapoda, Portunidae). Crustaceana. 90:1211–1233.

[CIT0005] Tan MH, Gan HM, Lee YP, Austin CM 2016 The complete mitogenome of the swimming crab Thalamita crenata (Rüppell, 1830) (Crustacea; Decapoda; Portunidae). Mitochondrial DNA Part A. 27:1275–1276. 10.3109/19401736.2014.945553 25090400

[CIT0006] Zhong SP, Zhao YF, Zhang Q 2018 The first complete mitochondrial genome of Dorippoidea from Orithyia sinica (Decapoda: Orithyiidae). Mitochondrial DNA Part B. 3:554–555. 10.1080/23802359.2018.1467237 PMC7800961 33474237

